# Efficacy and safety of scheduled rituximab in children with refractory nephrotic syndrome after multi-target therapy failure

**DOI:** 10.1007/s00467-025-07069-6

**Published:** 2025-12-12

**Authors:** Jing Yang, Yonghua He, Jinyun Pu, Yu Zhang, Jianhua Zhou, Liru Qiu

**Affiliations:** Department of Pediatrics, Hubei Provincial Key Laboratory of Pediatric Genetic Metabolic and Endocrine Rare Diseases, Hubei Provincial Clinical Research Center for Children’s Growth and Development and Metabolic Diseases, Tongji Hospital, Tongji Medical College, Huazhong University of Science and Technology, Jiefang Ave. No. 1095, Wuhan, 430030 China

**Keywords:** Rituximab, Refractory nephrotic syndrome, Multi-target therapy failure, Scheduled therapy, Children

## Abstract

**Background:**

Children with refractory nephrotic syndrome (NS) often experience frequent relapses despite combination immunosuppressive therapy. This study evaluated the efficacy and safety of scheduled rituximab (RTX) maintenance therapy in children with refractory NS who failed multi-target therapy.

**Methods:**

We retrospectively analyzed 48 children under 18 years old with steroid-dependent or steroid-resistant NS who had ≥ 2 relapses within 6 months despite corticosteroids plus at least two other immunosuppressants (multi-target therapy failure). Relapse rates before and after RTX, medication reduction, adverse events, and kidney outcomes were assessed.

**Results:**

Of 51 patients treated, 48 met inclusion criteria (three excluded for severe infusion reactions or loss to follow-up). RTX maintenance therapy significantly reduced the annual relapse rate from a mean of 2.1 relapses/year before RTX to 0.2 relapses/year after RTX (*p* < 0.0001). Over half of patients (56%) remained completely relapse-free during RTX treatment. Corticosteroids were successfully discontinued in 85% of patients (median 1 year after RTX), and all other immunosuppressants were stopped in 73% (median 0.8 years). Overall, 69% of patients were able to discontinue steroids plus two immunosuppressive drugs, maintaining remission on RTX alone. Adverse events were mostly mild: 56% had asymptomatic hypogammaglobulinemia, 8% experienced transient neutropenia (no agranulocytosis), 4% had mild infections, and 4% had infusion reactions (rash or throat discomfort) that resolved with supportive care. No patient progressed to kidney failure over a median follow-up of 2.5 years.

**Conclusions:**

Scheduled RTX maintenance therapy effectively maintained remission in children with multi-target therapy–refractory NS, allowing substantial reduction or cessation of steroids and other immunosuppressants.

**Graphical abstract:**

**Figure:**
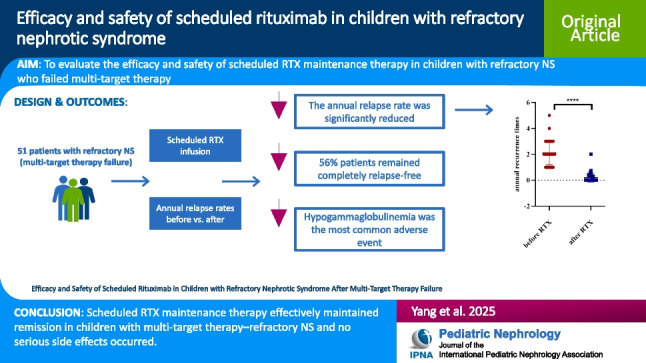
A higher resolution version of the Graphical abstract is available as Supplementary information

**Supplementary Information:**

The online version contains supplementary material available at 10.1007/s00467-025-07069-6.

## Introduction

Idiopathic nephrotic syndrome (NS) in children is characterized by edema, heavy proteinuria, hypoalbuminemia, hyperlipidemia, and a prothrombotic state. Its incidence varies widely by region and ethnicity, ranging from about 1.15 up to 16.9 per 100,000 children [1]. Prednisone is the cornerstone therapy for pediatric NS, and most patients achieve an initial steroid-sensitive remission. However, 30–50% of children experience frequent relapses or become steroid-dependent, requiring multiple courses of steroids [2, 3]. A smaller subset is steroid-resistant from the outset, failing to respond to adequate steroid therapy. For steroid-dependent NS (SDNS) and steroid-resistant NS (SRNS), clinicians often apply multi-target therapy—corticosteroids combined with other immunosuppressants such as calcineurin inhibitors (e.g., cyclosporine or tacrolimus) and cell-cycle inhibitors (e.g., cyclophosphamide, mycophenolate mofetil). In some refractory cases, two or more steroid-sparing agents are used concurrently alongside steroids. Despite these aggressive regimens, many patients continue to relapse frequently, incurring cumulative drug toxicities and morbidity. Long-term use of high-dose steroids and multiple immunosuppressants in childhood can lead to serious adverse effects, including growth failure, osteoporosis, infections, hypertension, and metabolic disturbances.

Rituximab (RTX) is a chimeric monoclonal antibody targeting the CD20 antigen on B-lymphocytes, leading to B-cell depletion [4]. Initially developed for B-cell malignancies (non-Hodgkin lymphoma), RTX has been successfully repurposed to treat various autoimmune diseases and glomerular disorders. Over the past 15 years, RTX has emerged as a promising therapy in difficult-to-treat pediatric NS. A landmark randomized controlled trial in children with frequently relapsing or steroid-dependent NS demonstrated that a single course of RTX significantly prolonged relapse-free survival and reduced maintenance immunosuppressant requirements compared to placebo [5]. Subsequent studies and meta-analyses have confirmed the efficacy of RTX in reducing relapses in both steroid-dependent and some steroid-resistant cases of NS. RTX can induce remission even in patients who have failed multiple other therapies [6]. However, the optimal dosing regimen and maintenance strategy for RTX in NS remain unclear. Relapses often recur once B-cells reconstitute after RTX, usually within 6–12 months, suggesting that scheduled maintenance infusions might be needed to sustain remission. There is limited data on long-term, scheduled RTX therapy in patients with the most refractory disease who have failed multi-target regimens. Here, we report our single-center experience using planned, repetitive RTX infusions (“scheduled RTX maintenance”) in children with refractory NS who had frequent relapses despite multi-target therapy. We specifically evaluate whether this approach reduces relapse frequency, permits withdrawal of steroids and other immunosuppressants, and is safe in the long term. We also compare outcomes between patients who were initially steroid-sensitive (but became dependent) and those who were steroid-resistant to determine if RTX is effective across these subgroups. Our aim is to provide evidence for an effective remission maintenance strategy in pediatric patients with multi-drug refractory NS.

## Methods

### Study design and patients

We performed a single-center cohort study of children with refractory NS treated with RTX at the Pediatric Nephrology Department of Tongji Hospital (Wuhan, China). The inclusion criteria were as follows: (1) age < 18 years; (2) diagnosis of steroid-dependent NS (frequently relapsing and steroid-sensitive) or steroid-resistant NS; (3) history of multi-target therapy failure, defined as continuing frequent relapses (more than 2 relapses in 6 months) despite treatment with prednisone (or equivalent corticosteroid) plus at least two other immunosuppressive agents; and (4) received at least two doses of RTX with a follow-up of ≥ 24 months after the first RTX dose. Patients were excluded if they had not received a multi-target therapeutic regimen prior to RTX, or if they experienced a severe RTX- related allergic reaction precluding further treatment. Between January 2013 and December 2022, 51 patients with refractory NS received RTX in our center. Of these, two patients who developed severe allergic reactions to RTX and one patient who was lost to follow-up were excluded from analysis. The remaining 48 patients comprise the study cohort.

The study was approved by the Ethics Committee of Tongji Hospital (TJ-IRB202507058), and written informed consent was obtained from the parents or guardians of all patients.

### Treatment protocol

All patients received RTX infusions as maintenance therapy to prevent NS relapses. RTX was administered initially at 375 mg/m^2^ intravenously over several hours. Patients were pre-medicated with ibuprofen, promethazine, and dexamethasone to reduce infusion reactions. RTX was given only when the patient’s disease was in remission (urine protein negative or trace) to ensure safety. Following the first dose, peripheral blood B-cell levels (CD19^+ count) were measured within 1 week to 1 month. If B-cells were not adequately depleted (target < 5/µL), an additional dose was considered. Subsequent maintenance RTX infusions were scheduled at approximately 3- to 6-month intervals to maintain continuous B-cell depletion, rather than waiting for a clinical relapse. The dosing for maintenance RTX (not infusion timing) was adjusted based on CD19^+ B-cell count recovery: if B-cells were 5–10/µL, RTX 100 mg/m^2^ was given; if 10–100/µL, RTX 200 mg/m^2^; and if > 100/µL, RTX 200–300 mg/m^2^ was given. During RTX therapy, corticosteroids and other immunosuppressants were systematically tapered and discontinued in patients who remained in remission. Immunosuppressive drugs used prior to RTX in this cohort included calcineurin inhibitors (cyclosporine or tacrolimus), mycophenolate mofetil, cyclophosphamide, and/or mizoribine, often in various combinations with steroids. After starting RTX, these drugs were reduced one by one over months if no relapse occurred, aiming for complete cessation of corticosteroids and at least two other immunosuppressants. RTX was then continued as the sole maintenance therapy. If a patient experienced a relapse while on RTX maintenance, standard therapy (high-dose steroids ± other agents as needed) was used to induce remission, and RTX dosing intervals were reassessed. We discontinued RTX after a year of discontinuation of hormones and immunosuppressants, when the patient’s urinary protein level stabilized.

### Definitions

Steroid-dependent NS was defined as two or more relapses during corticosteroid tapering or within 2 weeks of stopping steroids, or the need for ongoing low-dose steroids to prevent relapse. Steroid-resistant NS was defined as failure to achieve remission after 8 weeks of standard high-dose steroid therapy. Multi-target therapy refers to any regimen combining corticosteroids with two or more additional immunosuppressive drugs. Failure of multi-target therapy was defined as a relapse frequency > 2 times in 6 months (or > 4 times per year) despite adherence to the multi-drug regimen.

Relapse was defined clinically by recurrence of heavy proteinuria (≥ 3+ protein on urine dipstick for 3 consecutive days, with accompanying hypoalbuminemia/edema).

Remission was defined as nil or trace proteinuria for 3 consecutive days, typically corresponding to proteinuria < 4 mg/m^2^/hour or urine protein:creatinine ratio < 0.2.

Annual relapse rate before RTX was calculated for each patient as the total number of relapses divided by the number of years from NS onset to the first RTX treatment. The annual relapse rate after RTX was calculated as the total number of relapses after initiating RTX divided by the total post-RTX follow-up duration in years. Relapse-free survival was defined as the time from the first RTX dose to the first subsequent relapse (or to last follow-up if no relapse occurred). Patients were categorized into two subgroups based on initial steroid responsiveness (initial steroid-sensitive (steroid-dependent/frequently relapsing) vs. initial steroid-resistant) for subgroup analysis of outcomes. Kidney function was monitored by estimating glomerular filtration rate (eGFR) at each visit; progression to kidney failure was defined as eGFR < 15 mL/min/1.73 m^2^ or need for dialysis/kidney transplant.

### Statistical analysis

Data were analyzed using SPSS 26.0. Continuous variables are presented as mean ± standard deviation (SD) if normally distributed, or median with interquartile range (IQR) if non-normal. Categorical variables are presented as frequencies and percentages. For comparisons of relapse rates before vs. after RTX for the same patients, a paired Student’s *t*-test or Wilcoxon signed-rank test was used as appropriate. For unpaired comparisons between subgroups (steroid-dependent vs. steroid-resistant), we used the Mann–Whitney *U* test for continuous variables (given non-normal distribution of relapse counts) and chi-square or Fisher’s exact test for categorical variables. Kaplan–Meier analysis was planned to estimate relapse-free survival, but most patients remained relapse-free, precluding meaningful median survival estimation. A two-tailed *p*-value < 0.05 was considered statistically significant.

## Results

### Patient characteristics

From January 2013 to December 2022, 48 children with refractory nephrotic syndrome (36 boys and 12 girls) met inclusion criteria for this study. The cohort’s clinical characteristics are summarized in Table 1. The median age at initial NS diagnosis was 4.25 years (range 0.9–11.8 years), and the median age at first RTX administration was 12.0 years (range 5.3–15 years). The disease duration prior to starting RTX was long, with a median of 7.8 years (IQR 5–13 years) of relapsing illness despite multi-target therapy. By clinical classification, 34 patients (71%) had steroid-dependent NS (initially steroid-sensitive but frequently relapsing) and 14 patients (29%) had steroid-resistant NS. All patients had received multiple immunosuppressive treatments before RTX; most patients (85%) had been exposed to both a calcineurin inhibitor (CNI) and an antimetabolite (MMF or cyclophosphamide) in addition to prolonged steroids. Kidney biopsy was performed in 43 of 48 patients (89.6%). The most common pathological finding was focal segmental glomerulosclerosis (FSGS) in 18 patients (42%), followed by minimal change disease (MCD) in 13 (30%). Mesangial proliferative glomerulonephritis (MPGN) was seen in 12 patients (28%), and half of them had IgM deposits in the mesangium. Notably, the distribution of histopathology differed between steroid-response subgroups: among steroid-dependent patients, MCD was the predominant lesion (42% MCD vs. 32% FSGS), whereas FSGS was most frequent in the steroid-resistant group (66% FSGS, with no MCD cases). Despite these differences, all patients in both subgroups had a history of multiple relapses under combination therapy prior to RTX. At the time of the first RTX dose, 46 patients (96%) were in remission (proteinuria resolved) and 2 patients had persistent nephrotic-range proteinuria that had been resistant to all prior treatments.

**Table 1:** The clinical characteristics of patients with different pathological types

	Steroid-dependent type (*n* = 35)	Steroid-resistant type (*n* = 13)
Male (%)	77%	69%
Age of onset (years old)	4.25	5
Disease course before RTX (years)	6.729 ± 2.5944	4.808 ± 2.025
Pathology classification (*n* = 43)	(*n* = 31)	(*n* = 12)
MCD	13(42%)	0
FSGS	10(32%)	8(66%)
MPGN	5(16%)	2(17%)
IgMN	3(10%)	2(17%)
Annual number of recurrences	2.03 ± 1.014	2.36 ± 0.505

### B-cell depletion and treatment course

All 48 patients received at least two doses of RTX (range 2–18 doses) and achieved effective B-cell depletion (CD19^+ B-cells < 5/µL) after the initial infusion. RTX infusions were repeated at scheduled intervals (generally every 3–6 months) to maintain B-cell depletion as described in the Methods section. The total follow-up time after the first RTX treatment was a median of 2.5 years (range 2–7 years) for the cohort. Over this period, RTX was used as a maintenance therapy to prevent relapses while other immunosuppressive drugs were tapered. By last follow-up, the majority of patients were off corticosteroids and other medications, as detailed below.

### Relapse frequency before and after RTX

**Fig. 1:**
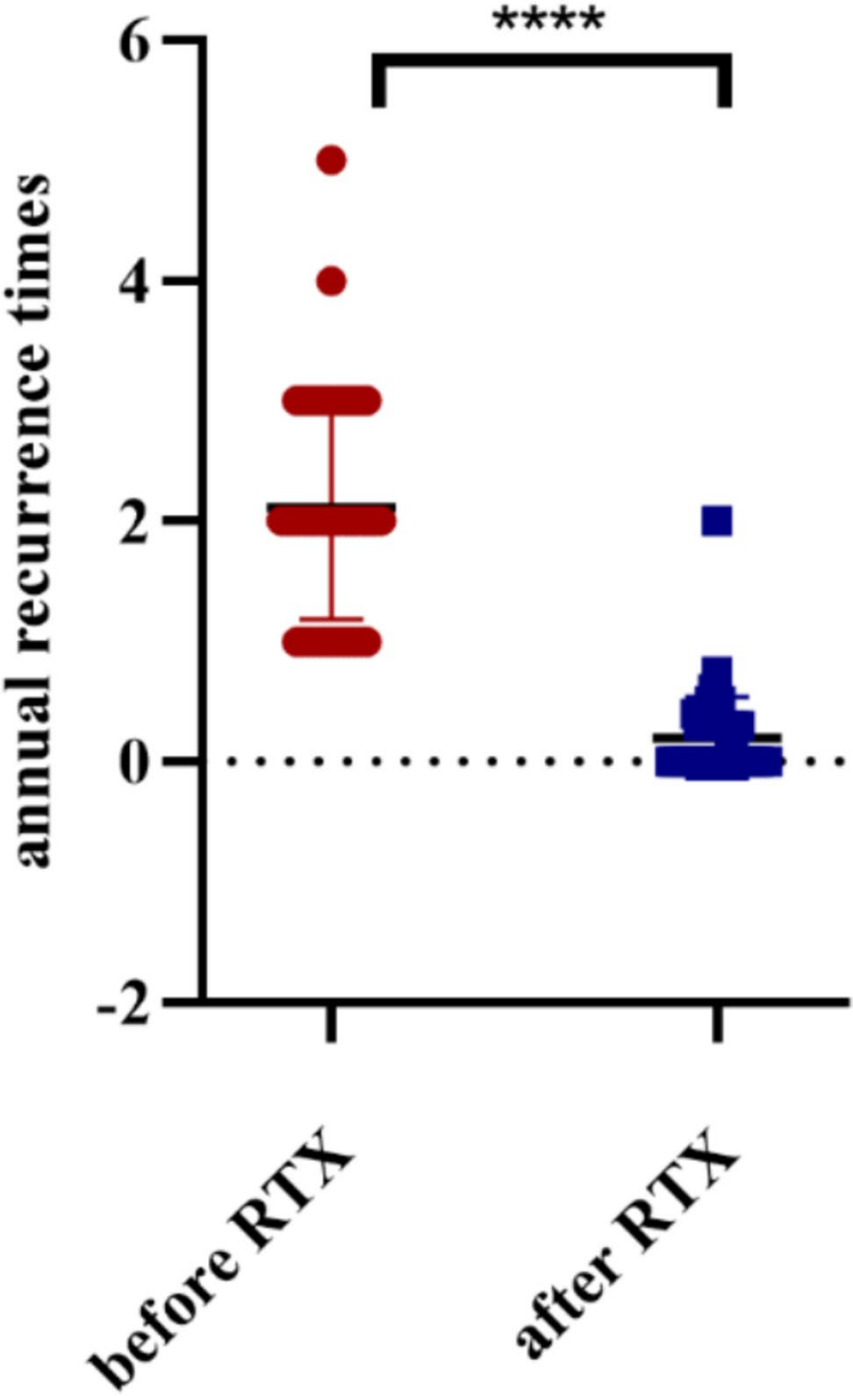
Annual relapse rates before and after RTX treatment in 48 children with refractory nephrotic syndrome. Each bar represents the mean (± SD) annual relapse frequency of the cohort. After initiation of scheduled RTX maintenance therapy, the mean relapse rate fell from ~ 2.1 relapses/year to ~ 0.2 relapses/year, a reduction of over 90% (**p < 0.0001). More than half of the patients experienced zero relapses during RTX treatment vs. frequent relapses prior to RTX

Before starting RTX therapy, patients experienced frequent relapses on multi-drug regimens. The mean annual relapse frequency prior to RTX (calculated among 46 patients who had any relapses; 2 patients with continuous proteinuria were not in remission at baseline) was 2.11 ± 0.92 relapses per year. After instituting scheduled RTX maintenance, disease control improved dramatically. A total of 27 out of 48 patients (56%) remained completely relapse-free throughout the RTX treatment period, and 21 patients (44%) experienced at least one relapse during follow-up. The overall relapse frequency dropped to a mean of 0.20 ± 0.34 relapses per year after RTX. This represents a highly significant reduction in relapse rate compared to the pre-RTX period (paired *t*-test, *p* < 0.0001; see Figure 1). Each individual patient saw a marked decrease in relapse frequency once on RTX, reflecting the effectiveness of RTX in maintaining remission.

**Fig. 2:**
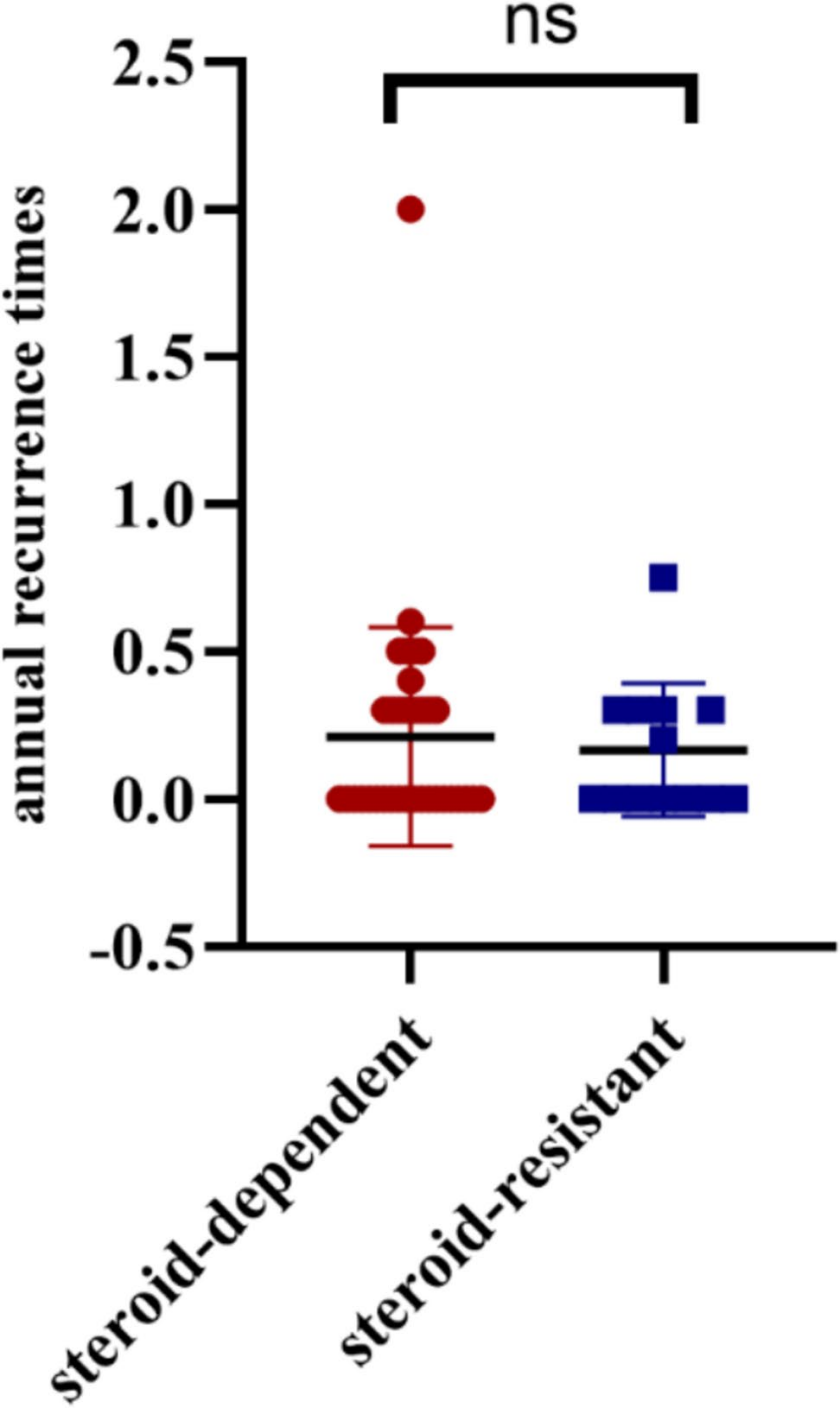
Comparison of post-RTX annual relapse rates in initial steroid-sensitive (steroid-dependent) vs. initial steroid-resistant patients. Each dot represents an individual patient’s relapse rate after RTX; horizontal lines indicate median values for each subgroup. There was no statistically significant difference in relapse frequency between steroid-dependent and steroid-resistant groups (ns = not significant), suggesting that RTX maintenance therapy was equally effective in reducing relapses in both populations

To further explore the efficacy of RTX across different patient subsets, we compared outcomes between the initial steroid-dependent and initial steroid-resistant groups. Both subgroups showed a significant decline in relapse frequency after RTX. Among the 34 initial steroid-dependent patients, 20 (59%) had no relapses on RTX, while 7 of 14 initial steroid-resistant patients (50%) achieved complete relapse-free status on RTX—a difference that was not statistically significant. The distribution of post-RTX annual relapse rates was similar in steroid-dependent vs. steroid-resistant cases (median 0 relapses/year in both groups). Patients who were steroid-resistant initially benefited from RTX maintenance nearly as much as those who were steroid-dependent. There was no significant difference in the post-RTX relapse rate between the two groups (*p* = 0.8474; see Fig. 2).

### Immunosuppressant discontinuation after RTX

One major goal of adding RTX was to spare or eliminate other immunosuppressive medications. The outcomes in this regard were very encouraging. After achieving sustained remission with RTX, 41 patients (85%) were able to successfully discontinue corticosteroids. The median time to complete steroid withdrawal was 1 year after starting RTX (some patients tapered off steroids within 6 months, while a few required up to 2 years to fully stop). Similarly, 35 patients (73%) discontinued all other immunosuppressive drugs (such as CNIs, MMF) during the RTX maintenance period, with a median time of about 0.8 years to come off these agents. By the end of follow-up, 33 patients (69%) had stopped corticosteroids plus at least two other immunosuppressants, effectively coming off their previous multi-target therapy entirely. These 33 patients were maintained in remission with RTX as the sole ongoing therapy. The remaining patients had at least one immunosuppressant (often a low-dose CNI or MMF) continued along with RTX, either due to physician caution or recurrent mild relapses. Overall, RTX treatment enabled a substantial reduction in drug burden: the vast majority of patients were free of daily steroids, and nearly three-quarters were free of any maintenance immunosuppressant aside from RTX. This represents a significant improvement in quality of life and a reduction in potential medication side effects for these patients.

### Adverse events and safety

Throughout the RTX treatment period, a total of 349 RTX doses were administered to the 48 patients over the study period (a median of 7 doses per patient, range 2–15 doses). Adverse events were recorded in 28 of 48 patients (58%), but most were mild or asymptomatic laboratory abnormalities. The most common observation was a reduction in serum immunoglobulin G (IgG) levels, noted in 27 patients (56%). Hypogammaglobulinemia in these cases was typically moderate (IgG level between 300 and 600 mg/dL) and not associated with severe infections; no patient required intravenous Ig replacement. Transient neutropenia occurred in four patients (8%) during RTX therapy. These cases of neutropenia were self-limited, and importantly, no instances of severe agranulocytosis (absolute neutrophil count < 500/µL) were seen in our cohort. Two patients (4%) developed mild infections (upper respiratory tract infections) during periods of B-cell depletion; all resolved with standard outpatient treatment, and there were no opportunistic infections or hospitalizations for infection. Infusion reactions were observed in two patients (4%) during RTX administration—these included urticarial rash and throat discomfort developing during the infusion. The infusions were paused and symptomatic treatment was given (antihistamines and steroids), after which the reactions subsided without progression to anaphylaxis. These two patients were able to complete their RTX infusions with slowing of the infusion rate. Notably, two other patients had experienced more severe allergic reactions (shortness of breath, hypotension) with their first RTX dose and were withdrawn from further RTX (these were the two excluded patients mentioned earlier). No other serious adverse events were reported. There were no cases of severe infusion reaction among the patients who continued RTX, no cases of Pneumocystis pneumonia, tuberculosis, or other serious infection, and no malignancies. Growth and development were tracked: patients who came off steroids showed catch-up growth in height, and no patient showed new onset diabetes or cataracts after stopping steroids. Overall, our results indicate that a maintenance RTX regimen, with appropriate monitoring, is safe in the pediatric NS population, with manageable side effects.

The most significant laboratory adverse effect was hypogammaglobulinemia, highlighting the need for periodic Ig level monitoring and infection vigilance during prolonged B-cell depletion therapy.

### Kidney function outcomes

Preservation of kidney function is a key long-term concern in refractory NS, especially in those with FSGS. In our study, no patient progressed to kidney failure during the follow-up period. All patients maintained stable kidney function (estimated GFR remained > 60 mL/min/1.73 m^2^ in all cases at last follow-up). Among the 18 patients with FSGS lesions on biopsy, none showed a decline in kidney function to the level requiring dialysis or transplantation. The ability of RTX to control proteinuria and relapses likely contributed to preventing further glomerular injury. A few patients had residual mild proteinuria (non-nephrotic range) or hypertension that was managed with ACE inhibitors and angiotensin receptor blockers, but overall kidney outcomes were excellent. These data suggest that RTX maintenance therapy, by reducing proteinuria and disease activity, can help stabilize or preserve kidney function in children with refractory NS, at least over the medium-term follow-up of this study.

## Discussion

In this single-center study, we investigated the role of scheduled maintenance RTX therapy in children with refractory nephrotic syndrome who had failed multi-target therapy. The patients we studied represent an extremely challenging subset of NS: on average, they had 7–8 years of disease with frequent relapses despite combinations of corticosteroids, calcineurin inhibitors, antiproliferative agents, and others. This cohort’s characteristics reflect known epidemiological patterns of difficult NS—most patients were boys (75%), with disease onset in early childhood (median ~ 4 years), which is consistent with the higher incidence of NS in young boys reported in the literature (male-to-female ratio ~ 2:1 in pediatric NS) [7–9]. The majority of our patients had either SDNS or SRNS. As expected, focal segmental glomerulosclerosis (FSGS) was the most common histopathology in those with initial steroid resistance, whereas MCD was more frequent among those who were initially steroid-responsive but frequently relapsing. Both FSGS and MCD are known to underlie refractory NS, and prior studies have shown that such patients often require aggressive therapies and still have high relapse rates. Although RTX is not a first-line treatment for idiopathic NS in children, accumulating evidence over the past decade has demonstrated its efficacy in reducing relapses in difficult cases [10–14]. RTX was first reported as a successful therapy for a child with steroid-dependent MCD in 2006 (Gilbert et al.), and since then, numerous case series and trials have supported its use in both SDNS and SRNS [15]. A multicenter randomized trial by Iijima et al. in 2014 provided high-quality evidence that RTX can significantly prolong remission in children with frequently relapsing or steroid-dependent NS [4]. Following RTX infusion, many patients can maintain remission for several months, allowing tapering of steroids and calcineurin inhibitors. However, because B-cell depletion by RTX is temporary, relapses often recur when B-cells return, necessitating repeated courses. The optimal RTX regimen (frequency and dosing of maintenance infusions) is still debated [16–19]. Some centers administer RTX only at relapse or when B-cells repopulate, whereas others, like ours, have explored fixed-interval dosing to preempt relapses. Our study is unique in focusing on scheduled RTX maintenance in a cohort that had exhausted multi-drug therapy options. We found that by dosing RTX approximately every 3–6 months to keep B-cells suppressed, we achieved a remarkable reduction in relapse frequency—over 90% reduction in annual relapses compared to the pre-RTX period—even in patients with the most refractory disease. More than half of the patients had zero relapses over a median of 2.5 years of RTX therapy, and the remainder had a vastly lower relapse rate than before. These outcomes are notably better than what is typically seen with standard therapies in similar populations, and they compare favorably to remission rates reported in meta-analyses of RTX in NS (for example, ~ 53% remission in refractory FSGS and 80% in MCD) [20, 21]. Our high remission rate may be attributable to the proactive RTX dosing strategy: by maintaining continuous B-cell depletion, we likely prevented many immunologic triggers of relapse that might occur when RTX is given more intermittently. Importantly, we found that RTX was effective in both steroid-dependent and steroid-resistant patients, with no significant difference in post-RTX relapse rates between these subgroups. Historically, steroid-resistant NS, especially with FSGS, is much harder to treat and often has worse outcomes. Some previous reports have suggested that patients with steroid resistance or multi-drug resistance are more prone to relapse even after RTX treatment [17, 18, 22–24]. For instance, factors like multidrug dependence, prior cyclophosphamide use, or younger age have been linked to earlier relapse post-RTX in observational studies. In contrast, our results indicate that when RTX is used as a continuous maintenance therapy, even initially steroid-resistant patients can achieve sustained remission rates comparable to those of steroid-dependent patients. This is an encouraging finding, as it suggests RTX can overcome some of the relapse propensity in the most resistant cases, possibly by a persistent immunomodulatory effect. It should be noted that our subgroup analysis may be limited by sample size, but the clear overlap in relapse outcomes supports the notion that RTX benefits extend across different NS phenotypes. This lack of difference is illustrated in Fig. 2, where both groups have a median of zero relapses per year on RTX. Another significant outcome of our study is the substantial sparing of other immunosuppressive medications. By using RTX, we were able to wean the vast majority of patients off prednisone and additional steroid-sparing drugs. About 69% of patients ultimately required no medications other than intermittent RTX infusions to remain in remission. This has profound clinical implications. Long-term high-dose prednisone can cause growth failure, Cushingoid features, obesity, diabetes, osteoporosis, and psychological effects in children. Calcineurin inhibitors carry risks of nephrotoxicity, hypertension, and cosmetic side effects (gum hypertrophy, hirsutism), while drugs like cyclophosphamide have gonadal toxicity and malignancy risk. By withdrawing these drugs, we potentially mitigated many cumulative toxicities. In our cohort, we observed catch-up growth and fewer steroid-related complications in patients off therapy. A recent multicenter trial by Iijima et al. (2022) found that continuing low-dose mycophenolate mofetil (MMF) after a single course of RTX significantly prolonged remission in complicated frequently relapsing NS [25]. That study advocated combination therapy (RTX plus MMF) to maintain remission. In contrast, our approach was nearly the opposite—we tapered and stopped MMF (and other drugs) during RTX therapy—yet still achieved durable remissions in most patients. This difference underscores that repeated RTX dosing can maintain remission even without other immunosuppressants in many cases. Our findings suggest that RTX monotherapy, given at regular intervals, may be sufficient for maintenance in a large proportion of refractory NS patients, obviating the need for concurrent daily immunosuppression. This is a promising strategy to reduce treatment burden and improve pediatric patient quality of life.

The safety profile of RTX in our study was consistent with previous pediatric NS reports [26]. We did not encounter any unexpected adverse effects from long-term RTX use. The most frequent issue was hypogammaglobulinemia, likely due to B-cell depletion affecting antibody levels. While we did not routinely replace IVIG in asymptomatic patients, this finding highlights the importance of monitoring immunoglobulin levels and being vigilant about infections. Reassuringly, even with low IgG, serious infections were not observed, possibly because many patients had residual IgG or maintained protective titers from prior vaccinations. Neutropenia has been reported with RTX, particularly in younger children, with one study citing a 9.6% incidence of late-onset neutropenia in pediatric NS [26]. Our incidence of neutropenia (8%) was similar, but none of our patients developed agranulocytosis or severe infection. All cases resolved spontaneously, and we did not have to discontinue RTX due to hematologic side effects. Infusion reactions are a known acute risk with RTX; we mitigated this with pre-medication and slow infusion rates. Two patients had mild allergic reactions that were managed conservatively, and they were able to continue RTX. It is important to note that two other patients with severe anaphylactic reactions were excluded after the first dose—this underscores that while rare (about 4% in our series), severe RTX allergy can occur and preclude further therapy. Overall, RTX maintenance was well tolerated, and no patient had long-term organ toxicity attributable to RTX. This safety profile is favorable when compared to the significant side effects associated with prolonged high-dose steroids and calcineurin inhibitors. Our findings align with other studies that have reported RTX as a relatively safe option in pediatric NS, with careful monitoring [24, 27, 28]. The scheduled RTX infusion we describe is time-limited rather than being used indefinitely. During the treatment period, no serious side effects were observed. In future studies, we need to conduct prospective research to determine the optimal duration of RTX use in children with NS and to assess its long-term safety.

Although our study focused on children who received RTX late—after multi-target therapy failure and long disease duration—our results (major relapse reduction and steroid/IS sparing) raise the question of whether earlier RTX could provide greater cumulative benefit. Randomized data in frequently relapsing/steroid dependent NS indicate that RTX given earlier can prolong remission and reduce maintenance immunosuppression [4–6, 16, 17, 25], and relapses often follow peripheral B-cell reconstitution at 6–12 months, supporting scheduled maintenance dosing [16–19]. In our cohort, RTX maintenance reduced annual relapses from 2.1 to 0.2 and enabled discontinuation of steroids in 85% and other IS agents in 73%, with an acceptable safety profile (hypogammaglobulinemia being the most common, and infrequent infections). These findings suggest that earlier RTX may be reasonable in selected patients with high relapse burden or drug toxicity; however, the present study cannot determine the optimal timing, and the long-term implications of prolonged B-cell depletion in children warrant caution. Prospective trials comparing earlier vs. deferred RTX (and RTX with/without concomitant MMF) are needed to define timing, dosing, and safety in pediatric NS.

Despite the positive outcomes, our study has several limitations. First, the sample size (48 patients), while sizeable for a single-center pediatric NS study, is still modest, and the results should be interpreted with caution. Rare adverse events might not be observed in a cohort of this size. Second, our study design was not a randomized trial but a retrospective-prospective observational study. All patients received RTX, and there was no control group of similar patients without RTX for comparison. This means we cannot definitively prove that RTX was solely responsible for the improved outcomes—however, given that these patients had a long prior history of frequent relapses on other therapies, the dramatic change after RTX is strongly suggestive of RTX’s efficacy. There is also a potential selection bias in that RTX was given when patients were in remission (after induction therapy), and we did not include patients who failed to achieve remission at all. This could bias the results toward better outcomes, since those with unremitting disease (in whom RTX might not have been tried) are not represented. Additionally, because we did not randomize patients to different RTX regimens, we cannot conclude the optimal frequency of RTX dosing; our 3–6 month interval was empirically chosen and may not be necessary for all patients (some may stay in remission longer after a dose). Another limitation is that we relied on historical relapse rates for the “before RTX” comparison, which could be subject to recall inaccuracies or varying follow-up durations. We attempted to mitigate this by calculating annualized relapse rates. In addition, we did not perform anti-drug antibodies. In future prospective work, we plan to incorporate drug-tolerant ADA assays and to correlate ADA status with CD19 kinetics, infusion tolerability, and any breakthrough relapses, to more definitively address immunogenicity. Lastly, being a single-center study from one region and predominantly a Chinese patient population, the generalizability of our findings to other settings or ethnic groups needs further confirmation.

Nonetheless, our study provides valuable evidence supporting the use of scheduled RTX maintenance therapy in refractory pediatric NS. The results show that even for children who have failed multiple other therapies, RTX can maintain remission in the majority and significantly lessen the need for steroids and other drugs. This has the dual benefit of better disease control and reduced medication toxicity. For clinicians managing difficult NS cases, RTX offers a steroid-sparing option that targets B-cells, which are believed to play a role in the pathogenesis of NS relapses (potentially through autoantibody production or antigen presentation). By periodically depleting B-cells, RTX may modulate the immune system enough to prevent relapses. Our approach of regular low-dose RTX top-ups may be especially useful in patients who relapse quickly after B-cell return. It is worth noting that the long-term effects of prolonged B-cell depletion in children are still being studied; careful follow-up is required to watch for late-onset adverse events like secondary malignancies or persistent infections.

Future research should focus on optimizing RTX-based strategies: for example, comparing fixed-schedule dosing vs. B-cell count–triggered dosing, or combining RTX with low-dose maintenance immunosuppressants vs. RTX alone. Given our positive experience, a controlled trial of RTX maintenance monotherapy in refractory NS would be ethical and informative. Additionally, identifying biomarkers for relapse (such as memory B-cell reconstitution or immunoglobulin levels) could help tailor RTX timing to individual patient needs.

In conclusion, our study demonstrates that children with multi-target therapy–resistant nephrotic syndrome can achieve sustained remission with a regimen of scheduled RTX infusions, greatly reducing their reliance on steroids and other immunosuppressants. This treatment strategy was effective in both steroid-dependent and steroid-resistant cases and showed an acceptable safety profile. Scheduled RTX maintenance appears to be a promising approach to improve outcomes in refractory pediatric NS. Larger multi-center studies and long-term follow-up will be important to confirm our findings and to ensure the safety of this approach, but our results provide a strong rationale for considering RTX maintenance in the most difficult NS patients.

## Conclusion

Scheduled maintenance therapy with rituximab significantly improves disease control in children with refractory nephrotic syndrome who have failed multi-target immunosuppressive regimens. RTX maintenance led to a marked reduction in relapse frequency and allowed the discontinuation of corticosteroids and other immunosuppressants in the majority of patients, thereby minimizing treatment-related toxicities. The efficacy of RTX was comparable in initially steroid-dependent vs. steroid-resistant patients, indicating broad utility across refractory NS subtypes. RTX was generally safe over a 2–7-year follow-up, with mostly mild adverse effects and no serious long-term complications observed. While this single-center study suggests that regular RTX infusions can sustain remission and stabilize kidney function in severe pediatric NS, these findings should be confirmed by larger, controlled studies. Our experience supports the incorporation of RTX into the management paradigm for difficult-to-treat NS, to maintain long-term remission and improve patient outcomes. With appropriate monitoring, scheduled RTX therapy offers a valuable option to reduce relapse burden and liberate children from the side effects of prolonged steroid and immunosuppressant use.

## Supplementary Information

Below is the link to the electronic supplementary material.

Graphical abstract (PPTX 119 KB)

## Data Availability

The original contributions presented in the study are included in the article. Further inquiries can be directed to the corresponding author.
