# Supplementary material for: Efficacy and safety of scheduled rituximab in children with refractory nephrotic syndrome after multi-target therapy failure
Source: Pediatr Nephrol. 2025 Dec 12;41(5):1361–70. doi: 10.1007/s00467-025-07069-6 (PMC13009123; doi:10.1007/s00467-025-07069-6)
Supplement: Supplementary file 1 — Graphical abstract (PPTX 119 KB) [file 467_2025_7069_MOESM1_ESM.pptx]

## Slide 1
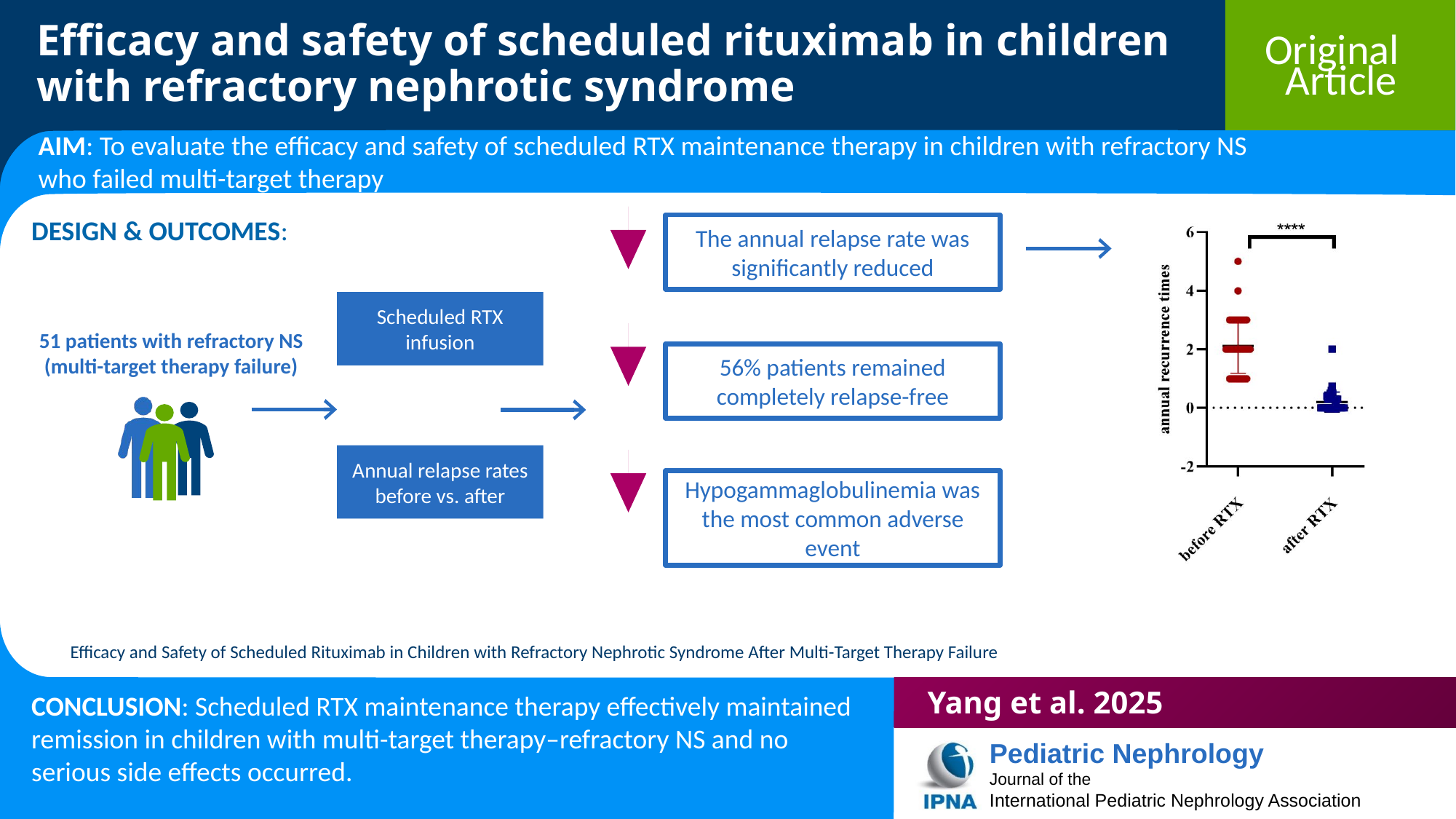

Efficacy and safety of scheduled rituximab in children with refractory nephrotic syndrome
AIM: To evaluate the efficacy and safety of scheduled RTX maintenance therapy in children with refractory NS who failed multi-target therapy
DESIGN & OUTCOMES:
The annual relapse rate was significantly reduced
Scheduled RTX infusion
51 patients with refractory NS
(multi-target therapy failure)
56% patients remained completely relapse-free
Annual relapse rates before vs. after
Hypogammaglobulinemia was the most common adverse event
Efficacy and Safety of Scheduled Rituximab in Children with Refractory Nephrotic Syndrome After Multi-Target Therapy Failure
Yang et al. 2025
CONCLUSION: Scheduled RTX maintenance therapy effectively maintained remission in children with multi-target therapy–refractory NS and no serious side effects occurred.
